# A method to predict edge strands in beta-sheets from protein sequences

**DOI:** 10.5936/csbj.201305001

**Published:** 2013-06-19

**Authors:** Antonin Guilloux, Bernard Caudron, Jean-Luc Jestin

**Affiliations:** a Analyse algébrique, Institut de Mathématiques de Jussieu, Université Pierre et Marie Curie, Paris VI, France; b Centre d'Informatique pour la Biologie, Institut Pasteur, Paris, France; c Département de Virologie, Institut Pasteur, Paris, France

**Keywords:** Protein structure, structure prediction, topology, beta-strands, central strand, loop, turn

## Abstract

There is a need for rules allowing three-dimensional structure information to be derived from protein sequences. In this work, consideration of an elementary protein folding step allows protein sub-sequences which optimize folding to be derived for any given protein sequence. Classical mechanics applied to this system and the energy conservation law during the elementary folding step yields an equation whose solutions are taken over the field of rational numbers. This formalism is applied to beta-sheets containing two edge strands and at least two central strands. The number of protein sub-sequences optimized for folding per amino acid in beta-strands is shown in particular to predict edge strands from protein sequences. Topological information on beta-strands and loops connecting them is derived for protein sequences with a prediction accuracy of 75%. The statistical significance of the finding is given. Applications in protein structure prediction are envisioned such as for the quality assessment of protein structure models.

## Introduction

Rules relating protein sequence and its three-dimensional structure are of special interest for protein structure prediction. Protein structures are mainly composed of beta-strands arranged in sheets, of helices and of loops and turns connecting them [1–3].

Beta-strands composing protein beta-sheets are bound either in parallel or in anti-parallel in particular by hydrogen bonds between amino acids’ main chain chemical groups [4–6]. Each beta-strand is bound to another two strands, except for the edge strands [7, 8]. Hydrophobic ordering plays an important role in the arrangement of amino acids and of beta-strands within beta-sheets. Hydrophobic side chains tend to be located centrally in the beta-sheet [9]. The more hydrophobic the beta-strand, the more centrally located is the beta-strand within the sheet [10]. The observation was found to be sufficient to account for beta-strand ordering in half of the beta-sheets and evidence for hydrophobic ordering was found in three-quarters of the beta-sheets [10, 11]. The length of beta-strands was also observed to be often smaller for edge strands [10]. Another rule was noted for four amino acids’ long strands: such beta-strands are central only if their hydrophilicity is smaller than 35% [12]. The last beta-strand in the protein sequence which is the closest to the protein C-terminus, was also found to be generally located at an edge for beta-sheets containing three to six strands [13]. Most three-stranded beta-sheets were found to be arranged in a sequential and anti-parallel order [14]. It was further reported that introduction of the positively charged amino acid lysine is sufficient to convert aggregating beta-strands within multimers into edge strands of monomers [15, 16]. Between two beta-sheets, interlocked pairs of beta-strands were identified as a common motif of protein structures [17, 18].

Protein structures were classified according to their fold [19–23]. The protein fold is straightforwardly derived from tertiary structures. While tertiary structure prediction from protein sequences remains a challenge for most proteins, their secondary structure is generally well predicted from their sequence [24–35]. Protein folding from a one-dimensional polypeptide chain into a three-dimensional compact protein globule was widely analyzed experimentally and theoretically [36–44].

An elementary protein folding step is defined here as the formation of a non-covalent bond between two atoms of the protein chain, such as a hydrogen bond. In this work, consideration of an elementary step of protein folding is shown to provide information on the three-dimensional structure from sequences.

## Experimental procedures

The programs pdb2 and pdb23 are written in perl. Their entry files are single PDB references of protein structures or lists of them [45, 46]. The program output files are tables (.xls files). The program removes DNA and RNA structures as well as those of peptides and proteins of less than 50 amino acids and analyzes only the first protein chain given in the DBREF key of the PDB file.

The program pdb2 uses the protein sequence in the three-letter amino acid code as found within the SEQRES key of.ent PDB files. From each.ent PDB file, a text.txt file contains the values of DBREF, SEQRES characterizing the protein sequences and the number of alpha carbon atoms (CA) within the PDB file so as to identify missing atoms within the structure. The mass of each atom is taken as the number of its nucleons, except for the selenium atom which was given the mass of a sulfur atom for the calculations, so as to avoid the bias due to selenomethionines deriving from methionine substitutions engineered for crystal diffraction studies. The protein sub-sequences were noted if their length does not exceed 20 amino acids (cf. results). L is the number of amino acids in the protein chain. For integers i within the 1 to L range, and j within the i to i+20 range, each sequence S(i,j) corresponding to the peptide from amino i to amino acid j is taken into account. If its mass M is not a square, the sequence S(i,j) is rejected. If its mass is a square, that is if the value M^1/2^ equals its integer part I(M^1/2^), then the sequence S is said to be optimized for folding (cf. results): S(i,j) = SOF(i,j). For all values of i and j associated to a protein, the set of all SOF(i,j) is drawn within a graph in red: Figure 3 shows the case of the human transthyretin protein of PDB reference 1eta.

Using the program pdb23 for any beta-sheet named (sheetID), the number (V) of SOF of the protein chain is given for each amino acid (AA) in the three-letter code in the downloadable output file together with the mean number (V_m_) of SOF per strand which is averaged over all amino acids of the beta-strand. To eliminate SOF sequences of length 1 corresponding to the unique amino acid cysteine, the SOF length was taken as (j-i) with its values in the range i+1 to j. Only beta-sheets with more than three beta-strands are taken into account (cf. discussion); beta-strands that are three or less amino acids long, are not considered within this analysis. Beta-sheets which do not contain edge strands such as those in beta-barrel structures have been excluded from this study.

Both programs can be used at the addresses (http://mobyle.pasteur.fr/cgi-bin/portal.py#forms::pdb2) and (http://mobyle.pasteur.fr/cgi-bin/portal.py#forms::pdb23).

The first training set of 29 structures (cf. supplementary material) was constituted by choosing one protein structure per fold in the SCOP database [22]. The two non-redundant test lists consisting of 83 protein structures from the PDB containing at least one open beta-sheet with more than three strands (cf. supplementary material) were established using the program check.pl by removal of proteins containing engineered substitutions within protein domains (except for the engineering of methionine to selenomethionine mutations whose impact for the calculation is described above). Proteins with similar functions and from similar organisms were also removed from the test sets. The all-alpha proteins found were further eliminated as they did not contain an edge strand within a beta-sheet. Protein homology within the test set was evaluated using the program Pisces [47]. The protein structures were visualized from pdbxxxx.ent PDB files using the software Pymol by highlighting their ribbon characterized by the amino acids’ alpha carbons.

## Results

**Figure 1:**
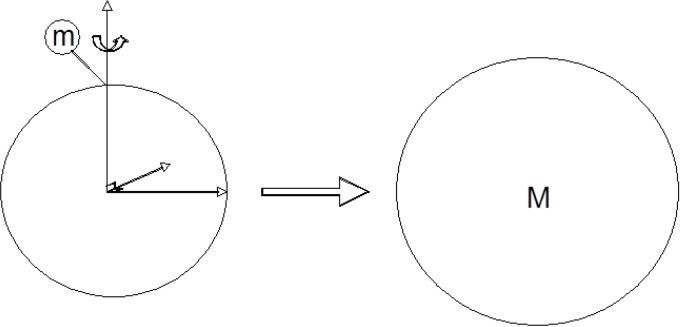
**E**lementary step for the folding of a small group of mass m onto the folding entity to yield a larger folding entity modelled by a sphere of mass M. Symmetry is gained during this elementary folding step.

A mechanical system consisting of a folding entity is modelled as a sphere (Figure 1). The reference frame is fixed with respect to the rotating folding entity so that its kinetic energy equals zero in this frame. A chemical group folding onto the folding entity is defined as the folding unit and is represented by a small sphere of mass m and velocity X. After folding, the folding entity is a larger sphere of mass M and velocity Y.

The kinetic energy of the folding unit is noted mX^2^/2. After folding, the kinetic energy of the larger folding entity is MY^2^/2. The internal energy released during folding is noted Ui. The difference in energy due to the breaking and the formation of bonds such as hydrogen bonds during the folding step is noted Ep. Energy conservation during the folding step can then be written as in equation (1):

$$\frac{\text{m}{\text{X}}^{2}}{2}=\frac{\text{M}{\text{Y}}^{2}}{2}+\text{E}$$

with E = Ui + Ep

**Figure 2:**
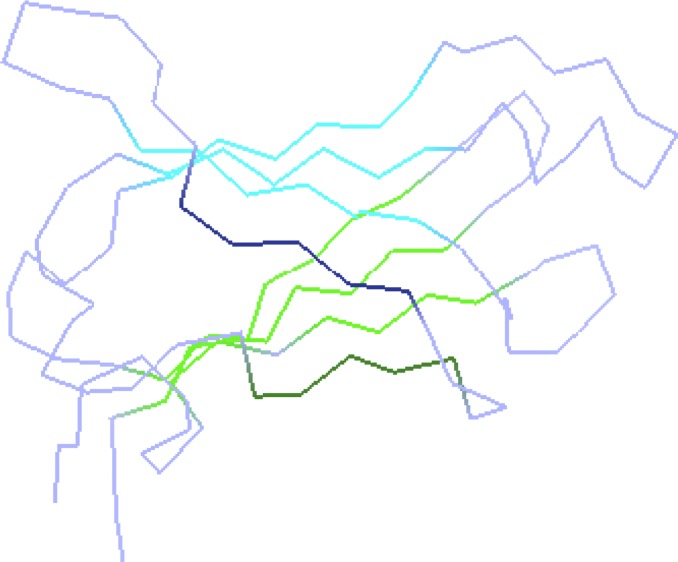
Representation of predicted edge strands in the structure of human transthyretin (PDB reference 1eta) [48]. Lines represent virtual bonds between the alpha carbons of adjacent amino acids in the protein. Two superimposed beta-sheets (blue and green) consisting of four beta-strands each contain two edge strands (dark blue and dark green) and predicted according to the rule.

Equation (1) is of special interest when considered over the field of rational numbers Q: for any given value of E, equation (1) has an infinite number of solutions in X and Y if (m/M) is a square (cf. Appendix). The folding of a mass m which is a square is further considered: for energy conservation to be ensured during the elementary folding step while having an infinite number of solutions in X and Y, it is sufficient for M to be a square. This condition prompted us to investigate the corresponding peptide sequences which are thereby optimized for folding. According to this model, if equation (1) has no solution in X and Y, then energy is not conserved during the elementary folding step and folding cannot proceed. Sets of protein sub-sequences with optimal folding properties (SOF) can be defined for any protein sequence. According to the elementary protein folding step (Figure 1), symmetry is gained during folding, as the small sphere of mass m on the surface of the folding entity yields a sphere after folding: the inequivalent group of mass m becomes equivalent to the other parts of the entity after folding. This formalism is used in this study to predict edge strands in protein beta-sheets (Figure 2).

**Figure 3:**
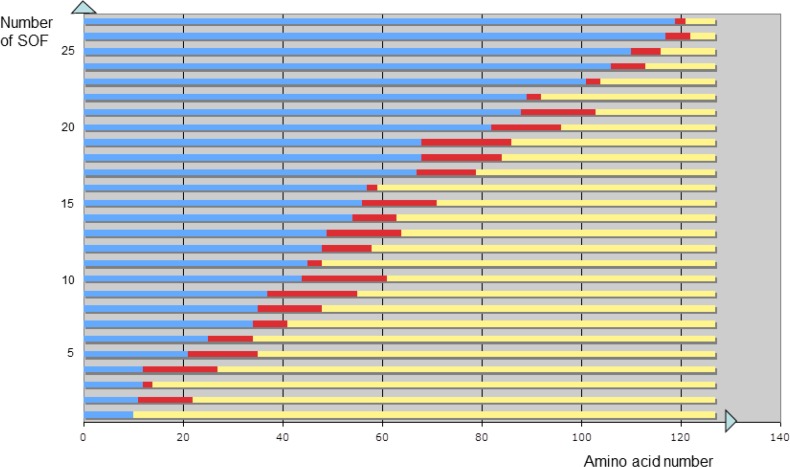
Set of sequences with optimal folding properties (SOF) highlighted in red for human transthyretin whose structure was described (PDB reference 1eta) [48]. The amino acid numbers are drawn on the horizontal axis. Each red segment corresponds to a peptide sequence with optimal folding properties (SOF).

The longer a sequence with optimal folding properties (SOF), the less stable it is upon amino acid substitution during evolution, given that the probability for an amino acid mutation to occur increases with the sequence length. Conversely, the shorter a SOF, the higher its robustness upon mutation. Accordingly, we did not consider SOF which are more than twenty consecutive amino acids long (Figure 3).

Edge strands are bound to a unique other beta-strand within beta-sheets while central strands are bound to two other beta-strands, thereby highlighting distinct symmetry properties. As the elementary folding step changes the symmetry of the system (Figure 1), we reasoned that sequences with optimal folding properties (SOF) might be correlated with the position of beta-strands within sheets located either centrally or on the edge.

By using a first training set of 29 proteins, a correlation was noted between extreme values of the mean number of SOF for a strand (V_m_) and its location on the edge of sheets of more than three strands. The following first rule was then established: the lowest value of V_m_ corresponds to an edge strand for (0 ≤ V_m_ < 0.34). If a V_m_ value does not exist in this range for all strands of the beta-sheet, the maximal V_m_ value predicts an edge strand (Table 1).

**Table 1:** Edge strands and central strands of the human transthyretin structure. Extreme values of the number of SOF (V_m_) highlighted in bold predict the edge strands noted in bold. The position of the strand in the sheet is central (C) or on the edge (E). Amino acids in the single-letter code are numbered according to the structure (PDB reference 1eta) [48].

Beta-strand	Position	V_m_
P11-D18	C2	1.9
G53-H56	**E2**	**4.3**
R103-S112	C1	1.1
S115-T123	E1	1
V28-A36	C1	3
A45-T49	**E1**	**3.6**
E66-D74	C2	2.8
H90-A97	E2	2.3

A first test set of 83 protein domain structures was made of protein structures with at least one open beta-sheet of at least four strands of more than three consecutive amino acids. Out of 96 predictions, 59 edge strands (61.5%) were predicted correctly.

As all beta-sheets considered in this study are composed of two edge strands and of at least two central strands, there is a probability of one half or less for the random assignment of an edge strand. In order to compute the *p* value of the test, the probability of failing at most k times within n assays (one assay for each protein beta-sheet) when the probability of failure is taken as 0.5 was computed using the binomial law as in Equation (2):

$$p=\frac{\sum_{\text{i}=0}^{\text{k}}{\text{C}}_{\text{n}}^{\text{i}}}{2^{\text{n}}}$$

where ${\text{C}}_{\text{n}}^{\text{i}}=\frac{\text{n}!}{\text{i}!(\text{n}-\text{i})!}$

The severity of this statistical test is highlighted by the fact that the probability of 0.5 is only exact for four-stranded beta-sheets, while it is less for the other beta-sheets of five strands or more considered in this work. For the first test set, the *p* value obtained for n = 96 and k = 37 was less than 0.0158 and was therefore considered as significant as it is less than 5%.

**Figure 4:**
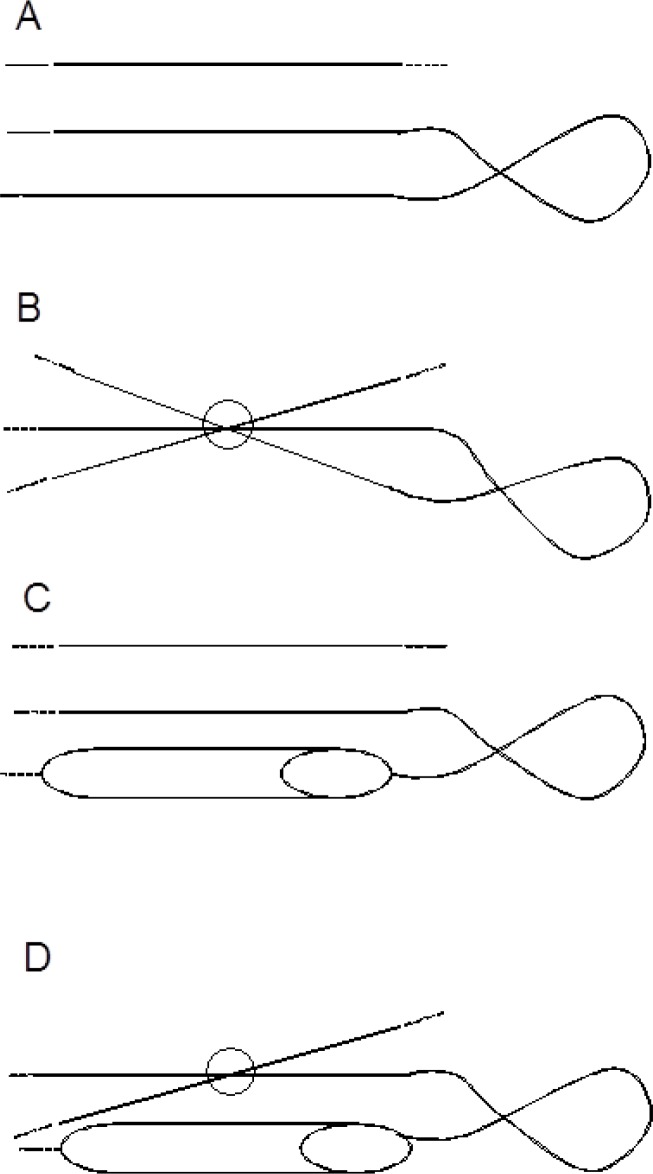
Bidimensional representations of polypeptide main chains containing 2D knots within loops between two beta-strands (A and B) or between a beta-strand and a helix (C and D). A beta-sheet's axis is represented by a small circle (B and D).

To improve the rule, the first test set was then used as the second training set in which the 37 structures associated to incorrect predictions of edge strands were further analyzed. It was noted that the rule is not valid anymore in case a central strand's end is bound to a two-dimensional knot (2D knot, Figure 4); the extreme V_m_ value is then associated to this strand or these strands, but not to an edge strand. The two-dimensional knot is defined here as the crossing of the polypeptide's main chain on a two-dimensional representation of the protein's structure along two axes, either the beta-sheet's axis (which crosses two alpha carbons within the first and last strands and minimizes the sum for all the strands of the distances of an alpha carbon per strand to the axis; Figure 4B and 4D) or the axis which includes the alpha carbon at the strand's end and which is perpendicular to the sheet's plane defined at proximity of the strand's end by two alpha carbon atoms at positions m and m+2 in the strand and by one alpha carbon of the paired amino acid in an adjacent strand (Figure 4A and 4C). The 2D knot is within a loop between a beta-strand and a helix or between two strands of the sheet considered (Figure 4). A 2D knot is not a three-dimensional knot in the polypeptide chain.

A second test set of 83 protein domain structures was then established to verify the improved rule (cf. supplementary material). 69 topological information predictions were found to be correct among the 92 predictions, thereby corresponding to a prediction accuracy of 75%. Use of equation (2) yielded a *p* value smaller than 8.4x10^−7^. This upper limit of the probability for at most 23 prediction failures by random assignments among 92 assays indicates the finding's statistical significance, which is far below the commonly accepted standard threshold of 0.05.

As an amino substitution within an edge strand was shown to alter beta-sheet aggregation, a link between protein solubility and correct predictions of topological information was further investigated. The prediction of protein solubility from their sequence had been widely investigated [49–54]. Using the protein solubility prediction program Proso II (http://mips.helmholtz-muenchen.de/prosoII/prosoII.seam), the second test set was found to be composed of 44 soluble proteins and 39 insoluble ones. The correct topological prediction rate of 75% was not found to be significantly different for soluble proteins (78%) and for insoluble proteins (74%).

The protein domains of the second test set were found to be distributed over the three major domains which are Bacteria (41 chains), Eukarya (36 protein chains from Animalia (25), from Fungi (5), from Plantae (4) and from Protista (2)) and Archaea (3 chains) and include three viral proteins. In this test set, biases were finally noted towards human proteins (about one fifth of the protein chains), pathogenic micro-organisms (about one sixth of the chains from seven species), *Escherichia coli* proteins (one sixth of the chains) and proteins from thermophilic bacteria (one tenth of the chains). The second test set with proteins chosen according to different functions and organisms was then analyzed by looking for potential sequence homology using the culling server Pisces [47]. Accordingly, four pairs of sequences were found to have more than 40% identity, namely (1na7, 1zog), (1gav, 2ms2), (1nxw, 2pl1), (2boi, 2chh) which are still considered as different topological predictions; even though two sequences may be highly homologous, their sequence differences can yield two distinct and correct topological predictions by identification of the two edge strands for example. The topological information prediction using the improved rule has a statistical significance which remains unaltered.

## Discussion

A major challenge in biological chemistry consists of the identification of relationships between protein sequences and their functions on genomic scales [55, 56]. While knowledge of a protein structure does not necessarily imply that a function can be identified for the protein, deciphering of protein domain structures remains of major interest and can provide clues for potential functions [57]. To circumvent expensive and time-consuming experimental techniques such as NMR or X-ray diffraction on protein crystals, promising approaches rely on computational biology, on the statistical analysis of known protein structures as well as on simulations of polypeptide chain dynamics [58]. Rules that relate the one-dimensional protein sequence and its three-dimensional structure properties were identified [9, 11, 16, 59–65]. The link between correlated mutations in multiple sequence alignments and interacting amino acids in the three-dimensional structure was extensively studied [66, 67]. Alignments of more than thousand well-chosen homologous protein sequences recently allowed the identification of a sufficiently large number of correlated mutations so as to decipher domain structures [68–70]. Three-stranded beta-sheets are generally arranged sequentially in anti-parallel [14]. These beta-sheets were not considered in this work which focussed on larger sheets of more than three beta-strands, first because of previously established rules [14], second because the statistical analysis carried out above would not be as straightforward as in Equation (2) (i.e. in the case of a three-stranded beta-sheet, the probability to identify an edge strand by random assignment is not one half or less) and third because the improved rule may not apply to three-stranded beta-sheets which were excluded during the analysis of the first training set.

In comparison to the topological information prediction accuracy of 75% described above, machine-learning approaches yielded edge strand prediction accuracies of 70% and 75.6% using support-vector machines [12, 71, 72]. Decision-tree algorithms allowed an 83% prediction accuracy to be obtained [12]. It should be of interest to combine different approaches to possibly improve further the edge strand prediction accuracy in protein beta-sheets. Interestingly, the notion of quasi-spherical random proteins was put in the context of natural proteins and introduced independently of this work [73]. The efficiency of the method used here shall be largely improved by applying it to several homologous sequences whose three-dimensional structures are expected to be similar.

Equations from classical mechanics are commonly treated over the field of real numbers. Using the field of rational numbers has been found of interest in different fields of the natural sciences connected to classical mechanics [74, 75]. It may constitute the basis for a new extension of theoretical chemistry [76]. In the field of genetic coding, substitution matrices made also use of discretized parameters such as p-adic integers or p-adic rational numbers [77–79]; it is of interest for the understanding of why the genetic code is the way it is. Importantly, the formalism described herein, i.e. the treatment of Eq.(1) was validated within the genetic code [74], providing thereby support for its application to proteins. In the field of biological chemistry, the genetic code is of special importance because of its quasi-universality within living organisms on earth for several billions of years [80, 81]. Experimentally, it has been the subject of numerous studies so as to develop applications in protein engineering [82–84]. Theoretically, Rumer noticed discrete symmetries linked to degeneracy in the genetic code [85, 86]. A rationale accounting for those discrete symmetries derived from the discrete nature of single-base mutations which have a major role in protein evolution [78, 79, 87, 88]. More recently, the codon arrangement in the genetic code was found to optimize kinetic energy conservation in polypeptide chains by considering the masses of the canonical amino acids: the formalism constituted by an equation from classical mechanics treated over the field of rational numbers was validated by the statistical significance of the codon arrangement within the genetic code [74].

The notion of protein sub-sequences with optimal folding properties (SOF) was introduced in this work. The elementary folding step allows the definition of criteria which are not necessary for folding, but which are sufficient to define protein sub-sequences with optimal folding properties. Edge strands are noticeable within beta-sheets as they are the only strands which pair with a unique other beta-strand; central strands generally pair indeed with two other beta-strands. Beta-barrel structures constitute an exception as they do not have edge strands, so that these structures were not considered here. The formalism suggested in this study allows the identification of sequences which optimize folding within proteins. Prediction of edge strands based on the consideration of the elementary folding step and of symmetry changes (Figure 1) is consistent with the fact that edge strands and central strands differ by their symmetrical properties with respect to neighbouring strands in protein beta-sheets. From our statistical analysis of hundreds of protein structures, we conclude that the formalism associated to the elementary folding step applied to given protein sequences allows information on the topology of their three-dimensional structure to be extracted.

It will be of interest to try and apply the same formalism to other secondary structure elements such as protein helices. The algorithm described herein to get topological information on beta-sheets from sequences for thousands of potential protein structure models provides a basis for a fast check of their quality. Applications within the critical assessment of techniques for protein structure prediction (CASP) are envisionned [89].

## Supplementary Material

A method to predict edge strands in beta-sheets from protein sequences

## Abbreviations

X and Y are velocities; m and M are respectively the masses of the folding unit and of the folding entity after the folding step; PDB is the Protein Data Bank; SOF is a Sequence Optimized for Folding; L is the number of amino acids of a protein chain (its length); *p* is the statistical *p* value as defined in equation (2).
