# Supplementary material for: A method to predict edge strands in beta-sheets from protein sequences
Source: Comput Struct Biotechnol J. 2013 Jun 19;7:e201305001. doi: 10.5936/csbj.201305001 (PMC3962219; doi:10.5936/csbj.201305001)
Supplement: A method to predict edge strands in beta-sheets from protein sequences [file CSBJ-7-e201305001_SM0001.pdf]

SUPPORTING INFORMATION

A method to predict edge strands in beta-sheets from protein sequences

Antonin Guilloux [a], Bernard Caudron [b], Jean-Luc Jestin [c,*]

[a]*Analyse algébrique, Institut de Mathématiques de Jussieu, Université Pierre et Marie Curie, Paris VI, France*
[b]*Centre d'Informatique pour la Biologie, Institut Pasteur, Paris, France*
[c]*Département de Virologie, Institut Pasteur, Paris, France*

* Corresponding author. Tel.: +33 144389496
E-mail address: jjestin@pasteur.fr (Jean-Luc Jestin)

Supplementary material

The first training set consisted of 29 proteins :
1c3g, 1cqy, 1hoe, 1wkh, 1job, 1mjc, 2ld9, 3n7h, 1m94, 2jwz, 1ps9, 2fm4, 2cx6, 2bly, 2lc5, 2k11, 1xn9, 1wkt, 1a1n, 1apw, 1ds9, 1em8, 1i4m, 1e0g, 1pu1, 4ubp, 1qk9, 1qm6, 1i3v.

The first test set consisted of 83 proteins :
1ah6, 1ahk, 1ap8, 1arz, 1auq, 1avp, 1b4q, 1b5s, 1bkf, 1b1a, 1btv, 1buz, 1cyu, 1cz4, 1d7a, 1dgq, 1dus, 1dzt, 1eta(2), 1eqy, 1ew0, 1f98, 1f8q, 1f9m, 1f25, 1fmf, 1g28, 1gfw, 1gnq, 1gui(2), *1gyg*(2), *1fqw, 1hh9*(3), *1hyq, 1llt, 1ipi, 1jlj, 1kpf, 1m2e, 1mac*(2), *1mom, 1n3r, 1nz0, 1npz, 1owl, 1pag, 1pcv, 1pdx, 1pmh*(2), *1pow*(3), *1prs, 1qfz, 1qg3(2), 1qtp(2), 1qy7, 1rph, 1sg0, 1szv, 1tr0, 1tsh, 1ufl, 1uuy, 1ux7(2), 1v2e, 1w0n*(2), *2a9s, 2aac, 2ahc, 2ai6, 2av5, 2bov, 2c9d, 2chs, 2cmg(2), 2cns, 2cvl, 2d4d, 2dt9, 2dyn, 2eey, 2hct, 2vbz, 2vp9.*

The second test set consisted of 83 proteins :
1aun, 1c2t, 1d7j, 1dr7, 1dzr(2), 1ewx, 1f2h, 1fm1, 1fsp, 1gav, 1gsu, 1gxt, 1h1d, 1hdk(2), 1hsl, 1ima(2), 1iyf, 1j3w, 1joe, 1jq5, 1k0k, 1kbq, 1kcm, 1kdt, 1lqj, 1na7, 1nks, 1nox, 1nvi, 1nxw, 1ny7(2), 1o6a, 1ogd, 1op4, 1otb, 1pkf, 1ppx, 1q2d, 1qhn, 1qjf, 1qr2, 1rsd, 1t4z, 1tfv, 1tjn, 1ugy, 1v1s(2), 1vc1, 1vf4, 1vsx, 1ww4(2), 1xja, 1xpw, 1yac, 1yxx, 1zny, 1zog, 1zyn, 2amu, 2bmx, 2boi(2), 2chh, 2cwz, 2dks, 2e9p, 2eeb(2), 2ffg, 2fw2, 2gkg, 2kwi, 2ms2, 2ofc, 2oxh, 2ozx, 2phe, 2pl1, 2pju(2), 2pr6, 2q3p, 2qdx, 2qh9, 2qie, 2v8m.

The number in parentheses after a PDB reference is the number of beta-sheets analyzed in the corresponding structure if there is more than one sheet analyzed.

Appendix

Statement :

For m and M, two strictly positive integers and E, a non-zero rational number. A rational number r is defined as $m/M = r^2$. X and Y are two rational numbers.
Then the equation ($mX^2 = MY^2 + E$) has an infinity of solutions in X and Y.

Proof :

$$mX^2 = MY^2 + E \iff r^2X^2 - Y^2 = E/M \iff (rX - Y)(rX + Y) = E/M$$

For each non-zero rational number t, one gets a rational solution :

$$\begin{cases} rX - Y = tE/M \\ rX + Y = 1/t \end{cases} \iff \begin{cases} X = (1/t + tE/M) / 2r \\ Y = (1/t - tE/M) / 2 \end{cases}$$

Note :
The statement is independent of the value of E, which is unknown during folding of a polypeptide chain. If m/M is not a square, then the existence of solutions depends on conditions on E, which can be further analyzed using p-adic numbers.
